# Heterozygous deletion of exon 17 of the *Kit* gene impairs mouse spermatogenesis by attenuating MAPK-ERK signaling

**DOI:** 10.1186/s40659-025-00609-2

**Published:** 2025-05-13

**Authors:** Siyuan Lin, Min Yang, Weipeng Zhu, Changqi Yang, Yaosheng Chen, Peiqing Cong, Xiaohong Liu, Zuyong He

**Affiliations:** State Key Laboratory of Biocontrol, School of Life Sciences, Sun Yat-sen University, Guangzhou, 510006 China

**Keywords:** *Kit*, Exon 17, Testicular development, Spermatogenesis

## Abstract

**Background:**

A splice mutation that causes skipping of exon 17 in the *KIT* gene is a major reason for the dominant white phenotype of pigs. Exon 17 of the *KIT* gene may be related to differences in testis size and sperm quality among different pig breeds. Investigating the effects of exon 17 of the *KIT* gene on spermatogonia differentiation and testicular development is essential for understanding the genetic causes of reduced fertility and semen quality in pigs. To better understand the effects of the splice mutation of *KIT* on porcine spermatogenesis, we described an exon 17 deletion mouse model (*Kit* ^D17/+^) constructed by simulating splice mutations in *KIT* for functional verification.

**Results:**

Deletion of exon 17 of *Kit* severely impaired the differentiation of spermatogonia and promoted the apoptosis of germ cells, resulting in testicular dysplasia and decreased sperm quality and male fertility. Further transcriptomic analysis revealed inhibited expression of genes involved in meiosis and spermatogenesis and attenuated MAPK-ERK signaling in the testicular tissues of *Kit* ^D17/+^ mice. The attenuated MAPK-ERK signaling caused by impaired Kit phosphorylation was confirmed by western blotting.

**Conclusions:**

Our study demonstrated that deletion of exon 17 of *Kit* severely impaired spermatogenesis and testicular development, leading to decreased semen quality and male fertility. These findings verified the function of exon 17 in the *Kit* gene and provide a theoretical basis for improving the semen quality of dominant white pigs through correction of the splice mutation of *KIT*.

**Supplementary Information:**

The online version contains supplementary material available at 10.1186/s40659-025-00609-2.

## Background

Dominant white is a commonly observed coat color phenotype in a variety of mammalian species, such as white Franches-Montagnes horses and Landrace and Yorkshire white pig breeds [1–3]. The dominant white phenotype in domestic pigs is mainly determined by two mutations in *KIT* — a duplication and a splice mutation [4, 5]. The duplication mutation is an approximately 560-kb tandem duplication that encompasses the entire *KIT* gene and its upstream and downstream regulatory regions, whereas the splice mutation is a G > A substitution at the first nucleotide of intron 17 in at least one of the *KIT* copies. The splice mutation causes skipping of exon 17, leading to the production of a KIT protein with impaired tyrosine kinase activity [4–6]. The *KIT* gene is a typical proto-oncogene that contains 21 exons located on chromosomes 4, 5 and 8 in humans, mice and pigs, respectively [7–9]. The KIT protein is a member of the class III receptor tyrosine kinase family and consists of three principal functional domains: the extracellular immunoglobulin-like domain, which is crucial for ligand binding; the transmembrane domain, which facilitates protein anchoring within the cell membrane; and the intracellular domain, which includes the tyrosine kinase activity essential for signal transduction [10–12]. The SCF/KIT interaction leads to receptor dimerization and the activation of kinase activity. This initiates a cascade of signal transduction pathways that are crucial for regulating cell proliferation, apoptosis, differentiation and migration, including the phosphatidylinositol 3-kinase (PI3-K), Src, Janus kinase/signal transducers and activators of transcription (JAK/STAT), phospholipase-C (PLC-γ) and mitogen-activated protein kinase (MAPK) pathways [13]. MAPK pathways have been described at multiple levels in distinct biological processes; however, much has yet to be discovered about the relationship between SCF/KIT and the MAPK pathway in spermatogenesis.

*Kit* mutations often cause pleiotropic effects [1, 3]. Mammals with loss-of-function mutations in the *Kit* gene often exhibit pigmentation defects and sterility, underscoring the crucial role of this gene in melanogenesis and gametogenesis [14–16]. In mice, *Kit* mutations can lead to modifications in coat color, anemia and male sterility [10]. Furthermore, studies have shown that, compared with Duroc pigs, dominant white pigs (Yorkshire pigs or Landrace pigs) present smaller testes, reduced sperm motility and lower sperm density [17–19]. These findings suggest that structural mutations in *KIT* may be important factors influencing porcine testicular development and spermatogenesis. Spermatogenesis is a highly complex and conserved process of cell division and differentiation that includes the proliferation and differentiation of spermatogonia, the meiosis of spermatocytes and the formation of spermatozoa [7]. The *Kit* gene plays a crucial role in the onset and maintenance of spermatogenesis [20]. Inhibition of Kit in prepubertal mice impairs the mitosis of differentiating type A spermatogonia [21]. Additionally, knockdown of *Kit* in spermatogonia leads to arrest of the cell cycle at the G2/M phase and adversely affects cell proliferation and viability [22]. However, the specific effect of the splice mutation of the dominant white *KIT* allele on spermatogenesis remains to be elucidated.

We hypothesized that the splice mutation in *Kit* may attenuate its signaling function and alter the expression of critical genes involved in spermatogenesis, leading to impaired testicular development and fertility.

In this study, using a previously created gene-edited mouse model with heterozygous deletion of exon 17 of *Kit* (*Kit* ^D17/+^), we systematically investigated the effects of the splice mutation of the dominant white *KIT* allele on mouse testicular development and spermatogenesis and the underlying regulatory mechanisms.

## Methods

### Mouse model

The gene-edited mouse model with heterozygous deletion of exon 17 of *Kit* (*Kit*^D17/+^) was created previously [23]. Briefly, a pair of sgRNAs targeting exon 17 of the *Kit* allele were designed and injected into zygote pronuclei along with Cas9 mRNA (Fig. S1A). The injected zygotes are transferred into the oviducts of surrogate recipient female mice to produce *Kit* exon 17-deleted pups. The *Kit*^D17/+^ mouse was characterized by a piebald phenotype and transcription of *Kit* mRNA without exon 17 (Fig. S1B, C). All the mice used in this study were bred in the SPF animal room of the Experimental Animal Center of Sun Yat-sen University. The mice were maintained on a 12/12 h light/dark cycle. All the experimental animal operations strictly followed the animal experiment scheme approved by the Animal Ethics Committee of Sun Yat-sen University (approval number: SYSU-IACUC-2023-B0617).

### Real-Time quantitative PCR (RT‒qPCR)

Total RNA was extracted using TRIzol Reagent (Thermo Fisher Scientific, USA) and quantified using a Thermo Fisher NanoDrop 2000c spectrophotometer. Next, 500 ng of total RNA was reverse-transcribed using the reverse transcription kit HiScript III RT SuperMix for qPCR (Vazyme, China) according to the manufacturer’s instructions. The cDNAs were subjected to real-time qPCR amplification using SYBR qPCR Master Mix (Vazyme, China) according to the manufacturer’s instructions. The data were analyzed using the 2^−ΔΔCt^ method, and the gene expression levels were normalized to those of the housekeeping gene β-actin. The primers used for RT‒qPCR are listed in Table S2.

### Protein extraction and Western blotting

The testes were carefully dissected using surgical scissors and subsequently placed into homogenization tubes. To each sample, 150–250 µL of RIPA lysis buffer containing PMSF was added per 20 mg of testicular tissue. The testicular tissue was homogenized and subjected to centrifugation at 14,000 × g for 15 min. The supernatants were collected, and protein concentrations were determined using a BCA protein detection kit (Beyotime, China). The protein samples were then loaded and separated on a 10% SDS‒PAGE gel, followed by transfer onto polyvinylidene fluoride (PVDF) membranes (Millipore, USA). Next, the membranes were blocked with TBST (Tris-buffered saline with Tween 20 detergent) buffer containing 5% skim milk at room temperature for 1 h, and then incubated with specific primary antibodies at 4 °C overnight and finally incubated with horseradish peroxidase (HRP)-conjugated secondary antibodies at room temperature for 1 h. The target proteins were visualized by the chemiluminescent signal generated through the catalytic action of the enhanced chemiluminescence (ECL) substrate (Fude, China) on HRP. The signals were detected using the GelView 6000 Pro Western Blotting Detection System (Bio-Rad, USA). The antibodies used for western blotting are listed in Table S1.

### Hematoxylin and Eosin (H&E) staining

Mouse testes and epididymides of different ages were fixed in 4% paraformaldehyde at 4 °C overnight. The fixed tissues were dehydrated, embedded in paraffin, and subsequently cut into 5 μm sections. After deparaffinization and rehydration, the sections were stained using the Hematoxylin & Eosin Staining Kit (Solarbio, China) according to the manufacturer’s protocol. The stained sections were then mounted with neutral resin and covered with a coverslip.

### Immunohistochemistry

Testicular tissue sections were first deparaffinized in xylene twice for 10 min each time. The sections were subsequently rehydrated through a graded ethanol series and sequentially passed through solutions of 100%, 95%, 80%, 70%, and 50% ethanol. After being washed in PBS for 5 min to remove any residual ethanol, the sections were subjected to antigen retrieval by heating in citrate buffer (pH 6.0) in a microwave oven. This process involved an initial high-power setting for 2 min, followed by a low-power setting for 20 min to expose antigenic sites. The sections were then washed in PBS three times for 5 min each and stained with an IHC detection kit (Abcam, UK) according to the manufacturer’s protocol.

### Immunofluorescence

The procedures used before antigen retrieval were similar to those used for immunohistochemistry. The sections were rinsed in PBS three times for 5 min each, followed by a 1-h incubation in 10% goat serum at 25 °C to block nonspecific antibody interactions. After blocking, the sections were incubated with primary antibodies at 4 °C overnight. The next day, the sections were washed in PBS three times for 5 min each and incubated with appropriate secondary antibodies at room temperature for 1 h in a dark room. After washing, the sections were stained with DAPI for counterstaining of the cell nuclei and mounted with antifade mounting medium (Solarbio, China) to protect the fluorescent signal. Information on the antibodies used for the immunofluorescent analysis is summarized in Table S1.

### TUNEL assay

Apoptotic cells in mouse testes were examined histologically using a colorimetric TUNEL apoptosis assay kit (Beyotime, China) according to the manufacturer’s protocol.

### Transcriptome sequencing (RNA-seq)

RNA was extracted, sequenced and analyzed using custom services provided by Gene Denovo Biotechnology Co. (Guangzhou, China). Briefly, total RNA was extracted from testicular tissues of *Kit* ^+/+^ and *Kit* ^D17/+^ mice using a TRIzol reagent kit (Invitrogen, USA) according to the manufacturer’s protocol. The RNA quality was checked using RNase-free agarose gel electrophoresis, and the mRNA was subsequently enriched by magnetic beads with Oligo (dT). The enriched mRNA was subsequently broken into short fragments via ultrasonication and reverse transcribed into cDNA using the NEBNext Ultra RNA Library Prep Kit for Illumina (New England Biolabs, USA). The purified double-stranded cDNA fragments were end repaired, one base was added, and the fragments were ligated to Illumina sequencing adapters. The ligation reaction mixture was purified with AMPure XP beads (1.0×) and amplified by the polymerase chain reaction (PCR). The resulting cDNA library was sequenced using an Illumina NovaSeq 6000 platform.

### Bioinformatics analysis

To obtain high-quality clean reads, raw reads containing adapters were filtered by fastp (version 0.18.0). After rRNA was removed, the remaining clean reads were further mapped to the reference genome using HISAT2. 2.4. The mapped reads were assembled using StringTie v1.3.1 via a reference-based approach. For each transcription region, an FPKM (fragment per kilobase of transcript per million mapped reads) value was calculated to quantify its expression abundance and variations using RSEM software. Principal component analysis (PCA) was performed with the R package gmodels (http://www.r-project.org/). Genes with a false discovery rate (FDR) less than 0.05 and an absolute fold change ≥ 1.5 were considered differentially expressed genes. Gene Ontology (GO) and Kyoto Encyclopedia of Genes and Genomes (KEGG) analyses were performed online using the OmicShare tools at www.omicshare.com/tools.

### Sperm quality measurement

The cauda epididymides from adult mice were harvested, dissected, and incubated in BWW medium (Solarbio, China) at 37 °C for 30 min. The sperm samples were collected, and the parameters of sperm motility were measured using a computer-aided sperm analysis (CASA) system.

### Statistical analysis

All data were analyzed using t tests or one-way ANOVAs with GraphPad Prism 9 software. All experiments were repeated at least three times. Differences were considered statistically significant when *P* < 0.05 (*p < 0.05, **p < 0.01).

## Results

### **Deleting exon 17 of***Kit***impaired the development of the mouse testis and epididymis**

**Fig. 1:**
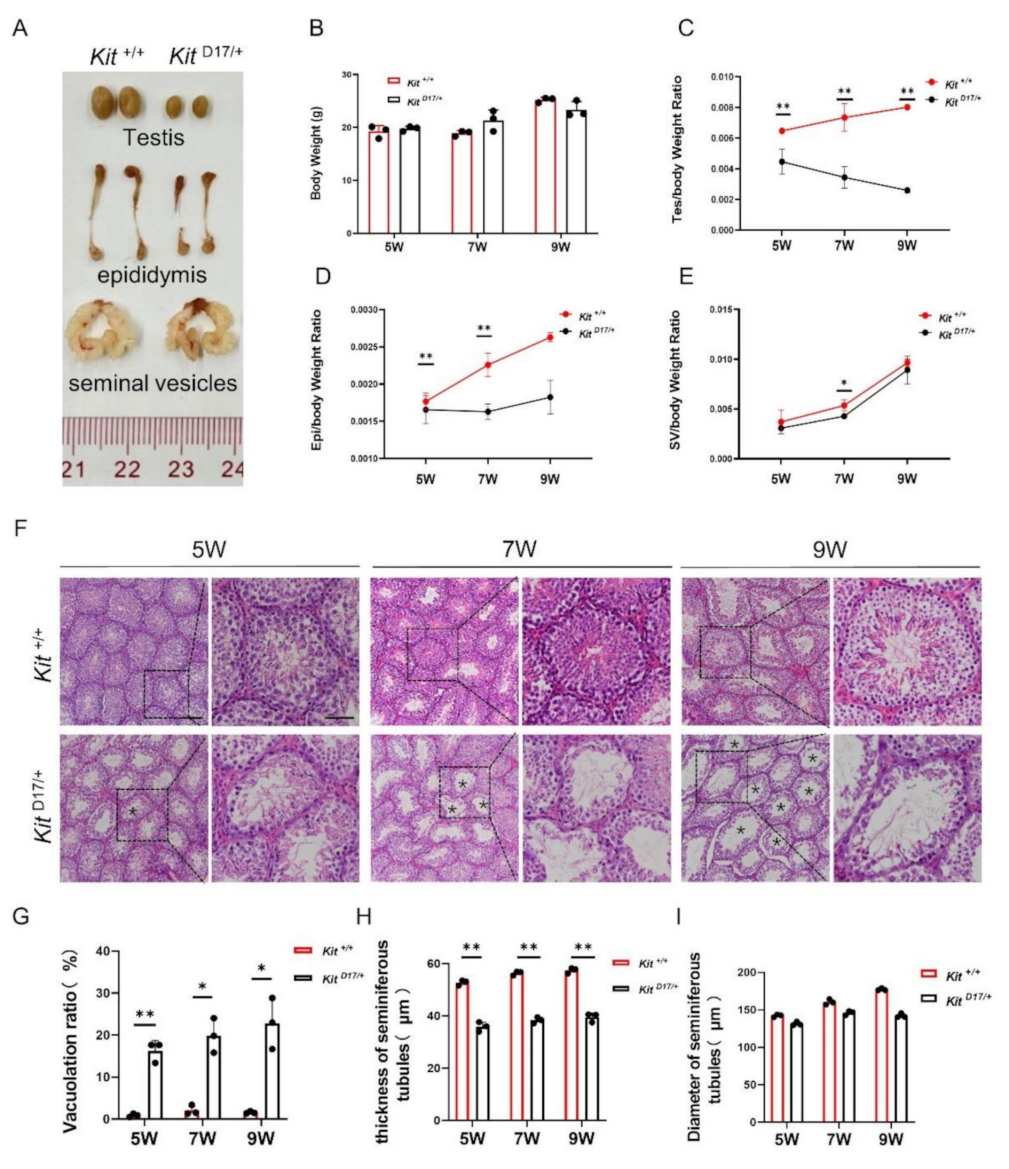
Deleting exon 17 of *Kit* impairs mouse testicular development. **(A)** Size of mouse testes, epididymes and seminal vesicles. **(B)** Individual weights of the mice. **(C)** The ratio of mouse testis weight to body weight. **(D)** The ratio of mouse epididymis weight to body weight. **(E)** The ratio of mouse seminal vesicle weight to body weight. *n* = 3, Student’s t test; **P < 0.01, *P < 0.05. **(F)** H&E staining of testis sections from 5-, 7- and 9-week-old mice. Scale bar = 50 μm (scale bar in magnified image = 30 μm). **(G)** Lumen vacuolization ratio. **(H)** Thickness of the seminiferous tubules. **(I)** Diameter of the seminiferous tubules. The data are presented as the mean ± SEM of three *Kit* ^D17/+^ and *Kit* ^+/+^ males, with 50 seminiferous tubule cross-sections analyzed per individual. Student’s t test; ^**^P < 0.01, ^*^P < 0.05

To assess the impact of deleting exon 17 of *Kit* on mouse reproductive organ development, we collected testes, epididymides, and seminal vesicles from mice at 5, 7 and 9 weeks (Fig. 1A). We did not observe a significant difference in body weight between *Kit* ^+/+^ and *Kit* ^D17/+^ mice at any age, suggesting that the deletion of exon 17 in *Kit* did not affect the normal growth of the mice (Fig. 1B). However, the testes from *Kit* ^D17/+^ mice were consistently smaller and lighter than those from age-matched *Kit* ^+/+^ mice across all time points, and the testis weight/body weight ratio revealed an increasing disparity with age between the two genotypes (Fig. 1A and C). By 9 weeks, the ratio in *Kit* ^+/+^ mice was nearly fourfold greater than that in *Kit* ^D17/+^ mice, indicating a significant effect of exon 17 deletion on testis growth relative to body weight (Fig. 1C). Additionally, the epididymides of *Kit* ^D17/+^ mice were notably smaller than those of *Kit* ^+/+^ mice, with the differences becoming more pronounced at 7 and 9 weeks of age (Fig. 1A and D). The differences in seminal vesicle weight between the two genotypes were notably subtler than weight differences in the testes and epididymides, suggesting that the impact of exon 17 deletion on seminal vesicle development was milder than its effects on the growth of the testes and epididymides (Fig. 1E). To elucidate the effects of exon 17 deletion in the *Kit* gene on the histological integrity of the testis, we conducted H&E staining. The analysis revealed a significant increase in the vacuolation ratio and a concurrent reduction in the thickness of the seminiferous epithelium in *Kit* ^D17/+^ mice, which could potentially impact spermatogenesis (Fig. 1G and H). Despite the observed structural changes, deletion of exon 17 in the *Kit* gene did not appear to influence the luminal diameter, suggesting that the impact on the seminiferous tubules is specific and does not extend to all aspects of tubular morphology (Fig. 1I).

### **Mice with***KIT***exon 17 deletion showed poor semen quality and reproductive ability**

**Fig. 2:**
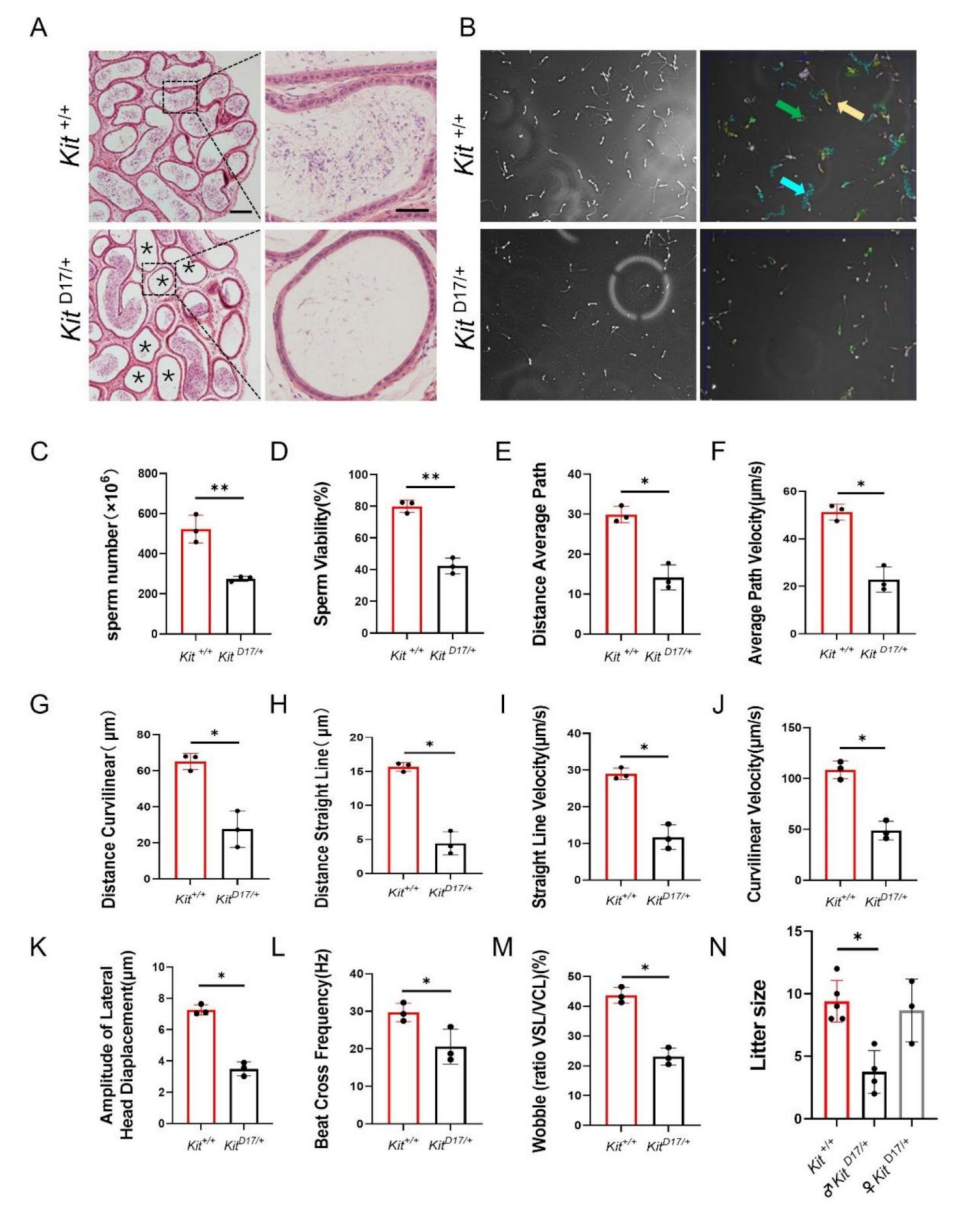
*Kit* ^*D17/+*^ mice presented lower sperm quality. **(A)** H&E staining of mouse epididymal sections. Scale bar = 50 μm (scale bar in magnified image = 30 μm), *n* = 3. **(B)** Mouse sperm number and sperm viability assay using the CASA system. The trajectories of the sperm were analyzed. Blue indicates fast trajectories, green indicates moderate-speed trajectories, and yellow indicates slow trajectories. Ten 1.5 s videos were recorded for each mouse. (C-M) Statistical graphs of sperm number, sperm viability, distance average path (DAP), average path velocity (VAP), distance curvilinear (DCL), distance straight line (DSL), straight line velocity (VSL), curvilinear velocity (VCL), amplitude of lateral head displacement (ALH), beat-cross frequency (BCF), and wobble (ratio of VSL/VCL). Student’s t test; **P < 0.01, *P < 0.05. (N) Litter sizes of (♂*Kit* ^+/+^ × ♀*Kit* ^+/+^), (♂ *Kit* ^D17/+^ × ♀ *Kit* ^+/+^), and (♂ *Kit* ^+/+^ × ♀ *Kit* ^D17/+^) mice, *n* ≥ 3. Student’s t test; **P < 0.01, *P < 0.05

Similar to that of the impaired structure of seminiferous tubules, the epididymides of *Kit* ^D17/+^ mice exhibited a vacuolized luminal structure with only a small amount of semen (Fig. 2A). Based on pathological changes in the testis and epididymis, we predicted that these changes would influence the sperm quality and reproductive ability of adult *Kit* ^D17/+^ males. Employing the CASA system, we assessed the parameters of sperm quality in *Kit* ^+/+^ and *Kit* ^D17/+^ adult mice. Compared with that of *Kit* ^+/+^ mice, the sperm number of *Kit*^D17/+^ mice was markedly lower (Fig. 2C). Additionally, sperm viability and other parameters such as average path velocity (VAP), curvilinear velocity (VCL), average path distance (DAP), curvilinear distance (DCL), straight line distance (DSL), straight line velocity (VSL), amplitude of lateral head displacement (ALH), beat cross frequency (BCF) and wobble (the ratio of VSL/VCL), were also significantly lower in *Kit* ^D17/+^ mice (Fig. 2D-M). Despite the lower sperm quality, *Kit* ^D17/+^ males were still fertile. However, the litter sizes of *Kit* ^D17/+^ males were considerably smaller than those of *Kit* ^+/+^ males (Fig. 2N). Interestingly, there was no significant difference in the litter size produced by wild-type and *Kit* ^D17/+^ female mice (Fig. 2N), suggesting that Kit signaling may be less critical for female reproductive development. In conclusion, deleting exon 17 of the *Kit* gene led to structural disruptions in both the testis and epididymis. This disruption resulted in a significant reduction in sperm count and motility, adversely affecting the reproductive ability of *Kit* ^D17/+^ males, as evidenced by the reduced litter sizes.

### **Deletion of exon 17 of the***Kit***gene inhibited spermatogonia differentiation**

**Fig. 3:**
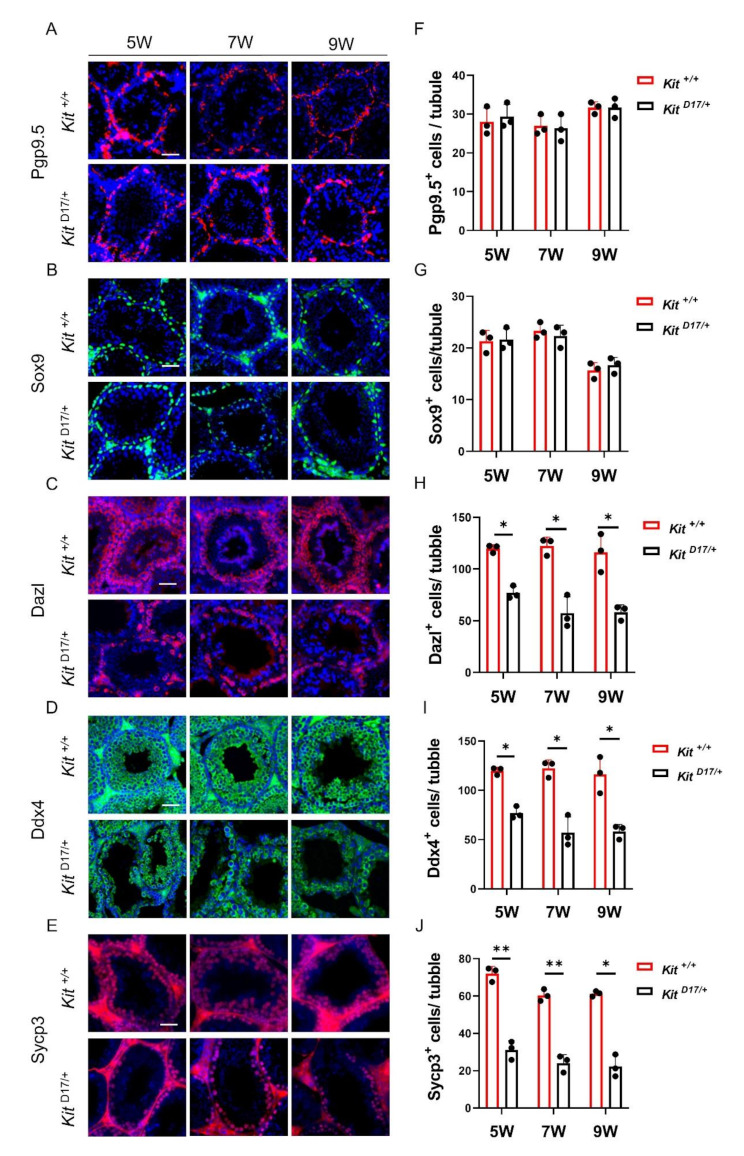
Deleting exon 17 of *Kit* impaired spermatogonia differentiation. Immunostaining of Pgp9.5^+^ **(A)**, Sox9^+^ **(B)**, Dazl^+^ **(C)**, Ddx4^+^ **(D)** and Sycp3^+^ **(E)** in testicular tissue sections from *Kit* ^D17/+^ and *Kit* ^+/+^ mice. Scale bar = 50 μm. **(F-I)** Quantitative analysis of the proportions of Pgp9.5^+^ **(A)**, Sox9^+^ **(B)**, Dazl^+^ **(C)**, Ddx4^+^ **(D)** and Sycp3^+^ **(E)** cells in testicular tissue sections from *Kit* ^D17/+^ and *Kit* ^+/+^ mice. The data are presented as the mean ± SEM of *Kit* ^D17/+^ and *Kit* ^+/+^ males, with 50 tubule cross-sections analyzed per individual, *n* = 3. Student’s t test; **P < 0.01, *P < 0.05

To further investigate which types of testicular cells were affected by the deletion of exon 17 of *Kit*, we conducted immunostaining on testicular tissue sections with cell type-specific markers. Pgp9.5 is widely used as a marker of undifferentiated spermatogonia, and Dazl, which is expressed in spermatogonia and spermatocytes in the testis, is utilized to assess the differentiation of spermatogonia and meiosis [24, 25]. Ddx4, a germ cell-specific protein, serves as a general germ cell marker [26], whereas Sycp3, one of the main components of the homologous chromosome synaptonemal complex, is predominantly localized between homologous chromosomes in spermatocytes undergoing meiosis [27]. Sox9 was used as a Sertoli cell marker [28]. The immunostaining results showed no change in the number of Pgp9.5^+^ and Sox9^+^ cells (Fig. 3A-D), suggesting that deleting exon 17 of *Kit* did not affect the formation or maintenance of undifferentiated spermatogonia or Sertoli cells. Conversely, a significant reduction in Dazl^+^, Ddx4^+^ and Sycp3^+^ cells was observed in *Kit* ^D17/+^ testes at 5, 7 and 9 weeks (Fig. 3E-I), indicating that exon 17 deletion inhibited the differentiation of spermatogonia.

### **Deleting exon 17 of***Kit***promoted germ cell apoptosis in mouse seminiferous tubules**

**Fig. 4:**
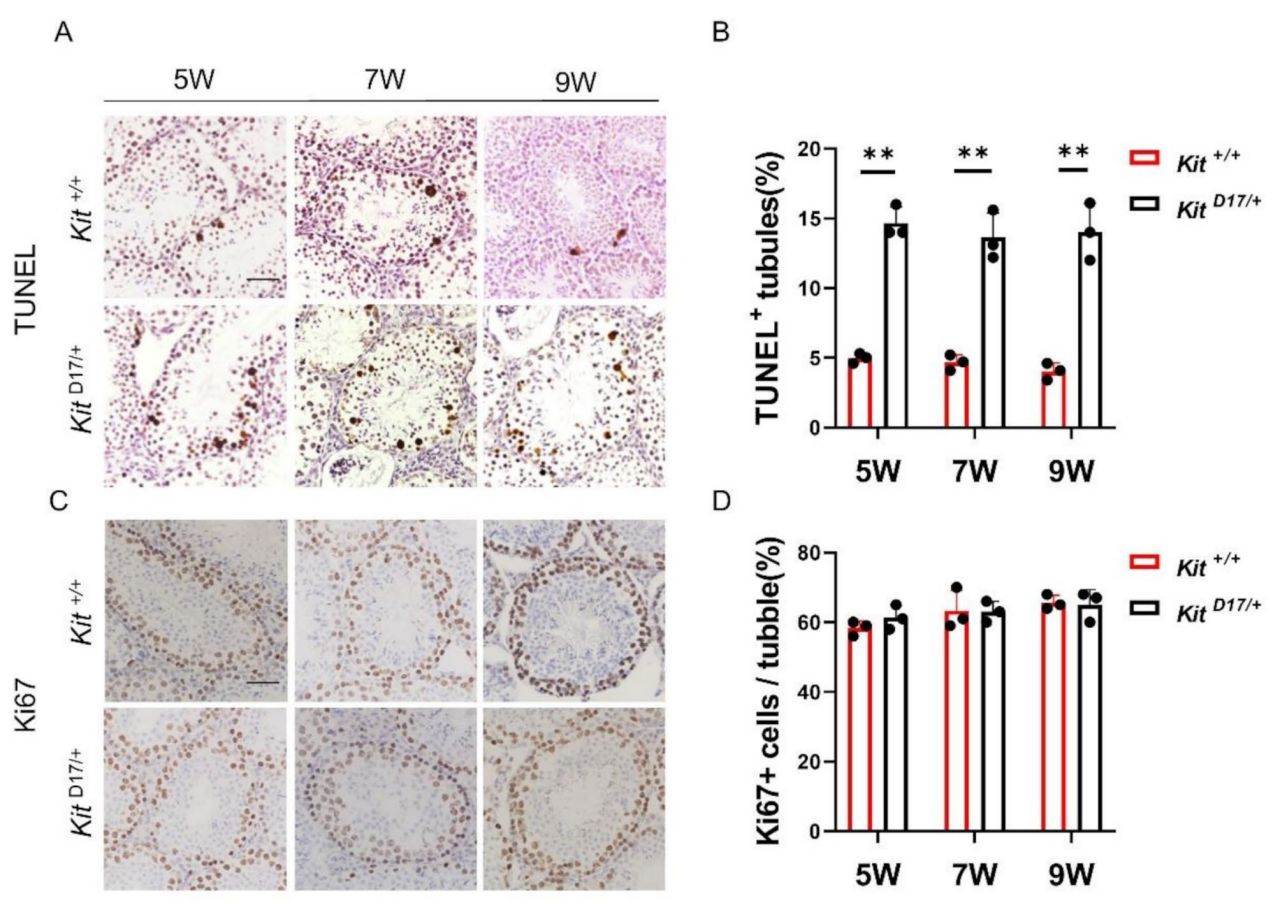
Deleting exon 17 of *Kit* promoted cell apoptosis in testicular tissues. **(A)** TUNEL assay to detect apoptotic cells in the testes of *Kit* ^D17/+^ and *Kit* ^+/+^ mice at 5, 7 and 9 weeks. Scale bar = 50 μm. **(B)** Quantitative analysis of the proportion of apoptotic cells in the seminiferous tubules of *Kit* ^D17/+^ and *Kit* ^+/+^ mice at 5, 7 and 9 weeks. **(C)** Immunostaining of Ki67 in the seminiferous tubules of *Kit* ^D17/+^ and *Kit* ^+/+^ mice at 5, 7 and 9 weeks. Scale bar = 50 μm. **(D)** Quantitative analysis of Ki67^+^ cells per tubule in the testes of *Kit* ^D17/+^ and *Kit* ^+/+^ mice at 5, 7 and 9 weeks. The data are presented as the mean ± SEM of three *Kit* ^D17/+^ and *Kit* ^+/+^ males, with 50 tubule cross-sections analyzed per individual. Student’s t test; **P < 0.01, *P < 0.05

As spermatogenesis was severely inhibited and fewer spermatogenic cells were observed in the seminiferous tubules of *Kit*^*D17/+*^ mice, we speculated that the deletion of exon 17 of *Kit* potentially affects cell proliferation or apoptosis in the seminiferous tubules. Therefore, a TUNEL assay was performed to investigate the impact of exon 17 deletion on the apoptosis of germ cells. The results showed a notable increase in apoptotic cells in the seminiferous tubules of *Kit* ^D17/+^ mice at 5, 7 and 9 weeks, with approximately 15% of tubules being TUNEL positive, indicating increased cell apoptosis caused by exon 17 deletion (Fig. 4A and B). The effect of exon 17 deletion on cell proliferation was further assessed by immunofluorescence staining of Ki67. The results showed no significant difference in the number of Ki67-positive cells between *Kit* ^+/+^ and *Kit* ^D17/+^ testes (Fig. 4C and D). In conclusion, deleting exon 17 of the *Kit* gene promoted the apoptosis of testicular cells but did not appear to affect their proliferation.

### **Inhibited expression of genes involved in meiosis and spermatogenesis in testicular tissues of***Kit*^**D17/+**^**mice revealed by transcriptomic analysis**

To further investigate the underlying molecular mechanisms of impaired spermatogenesis caused by deleting exon 17 of *Kit*, RNA-seq analysis of testicular tissues from *Kit*^+/+^ and *Kit*^*D17/+*^ mice at 5 and 7 weeks of age was carried out. Three independent RNA-seq experiments were performed on three pairs of *Kit* ^+/+^ and *Kit* ^*D17/+*^ testes. Unfortunately, three samples (5 W-WT-1, 5 W-D17-1, and 7 W-D17-3; WT and D17 represent *Kit* ^+/+^ and *Kit* ^D17/+^, respectively) did not pass the quality control, resulting in outliers for samples from the same time point and genotype in the PCA. Outlier samples increase the overall variability of the data, thereby affecting the identification of differentially expressed genes. Therefore, we removed the outlier samples, leading to the inclusion of two biological replicates for 5 W-WT, 5 W-D17, and 7 W-D17, and three biological replicates for 7 W-WT in the subsequent analysis (Fig. S2A).

**Fig. 5:**
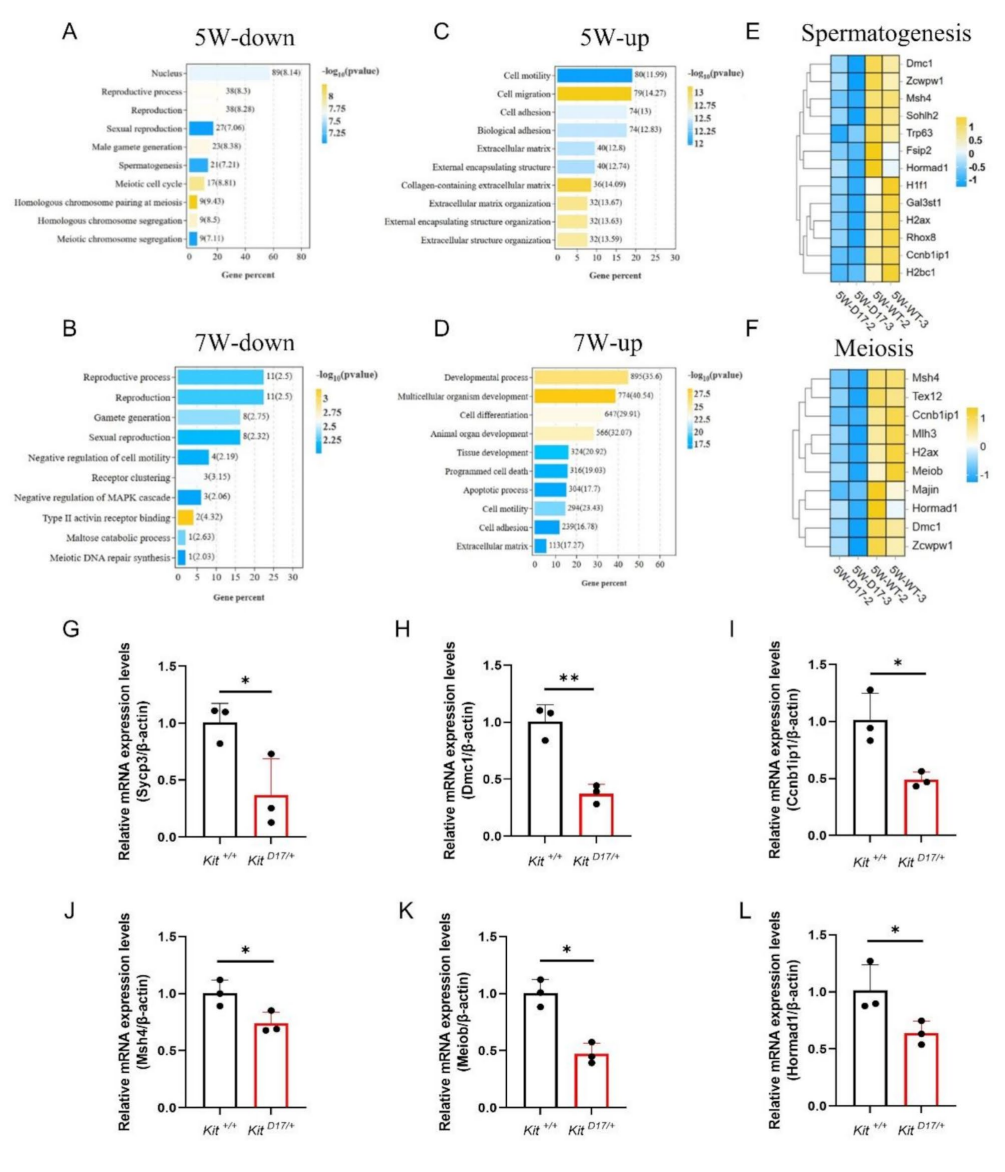
Gene ontology analysis and RNA-seq validation by RT‒qPCR analysis. (**A** and **C**) Gene Ontology enrichment of biological processes associated with the down- and upregulated DEGs between testicular tissues of *Kit* ^+/+^ and *Kit* ^*D17/+*^ mice at 5 weeks of age. (**B** and **D**) Gene Ontology enrichment of biological processes associated with the down- and upregulated DEGs between testicular tissues of 7-week-old *Kit*^+/+^ and *Kit* ^*D17/+*^ mice, *n* = 2 or 3. **(E)** Heatmap of spermatogenesis-related gene expression. **(F)** Heatmap of meiosis-related gene expression. **(G-L)** RT‒qPCR validation of DEGs, *n* = 3. Student’s t test; **P < 0.01, *P < 0.05

Among all the genes with altered expression profiles in the testicular tissues of *Kit* ^*D17/+*^ mice, 435 genes were upregulated (approximately 73%) and 162 genes were downregulated (27%) in 5 W-D17 compared with 5 W-WT, whereas in 7 W-D17, 2073 genes were upregulated (97.5%) and 53 genes were downregulated (2.5%) compared with 7 W-WT (Fig. S2B). When the DEGs for 5 W and 7 W (5 W-WT vs. 5 W-D17 and 7 W-WT vs. 7 W-D17) were subjected to GO analysis, we observed that downregulated genes in both age groups predominantly participated in the processes of reproduction, spermatogenesis and meiosis (Fig. 5A and B), which was consistent with the impaired spermatogenesis and differentiation observed in *Kit* ^*D17/+*^ mice. The upregulated genes were associated primarily with key biological processes such as cell adhesion, the extracellular matrix and cell motility (Fig. 5C and D). We further focused on the downregulated genes associated with spermatogenesis. We enriched a total of 17 genes related to spermatogenesis, including 10 genes with known specific functions, such as the double-stranded DNA break repair-related genes *Msh4*, *Mlh3*, and *Zcwpw1*; the meiotic homologous recombination-related genes *Dmc1*, *Meiob* and *Hormad1*; and the meiosis-related genes *Tex12*, *Ccnb1ip1*, *Majin*, and *H2ax* (Fig. 5E and F). The RNA-seq data were confirmed via RT‒qPCR of 6 genes (*Sycp3*, *Dmc1*, *Ccnb1ip1*, *Msh4*, *Meiob* and *Hormad1*) known to be involved in spermatogenesis (Fig. 5G-L). Overall, our transcriptomic analysis revealed the critical role of exon 17 of the *Kit* gene in the regulation of spermatogenesis.

### **Attenuation of MAPK-ERK signaling in testicular tissues of***Kit*^**D17/+**^**mice**

**Fig. 6:**
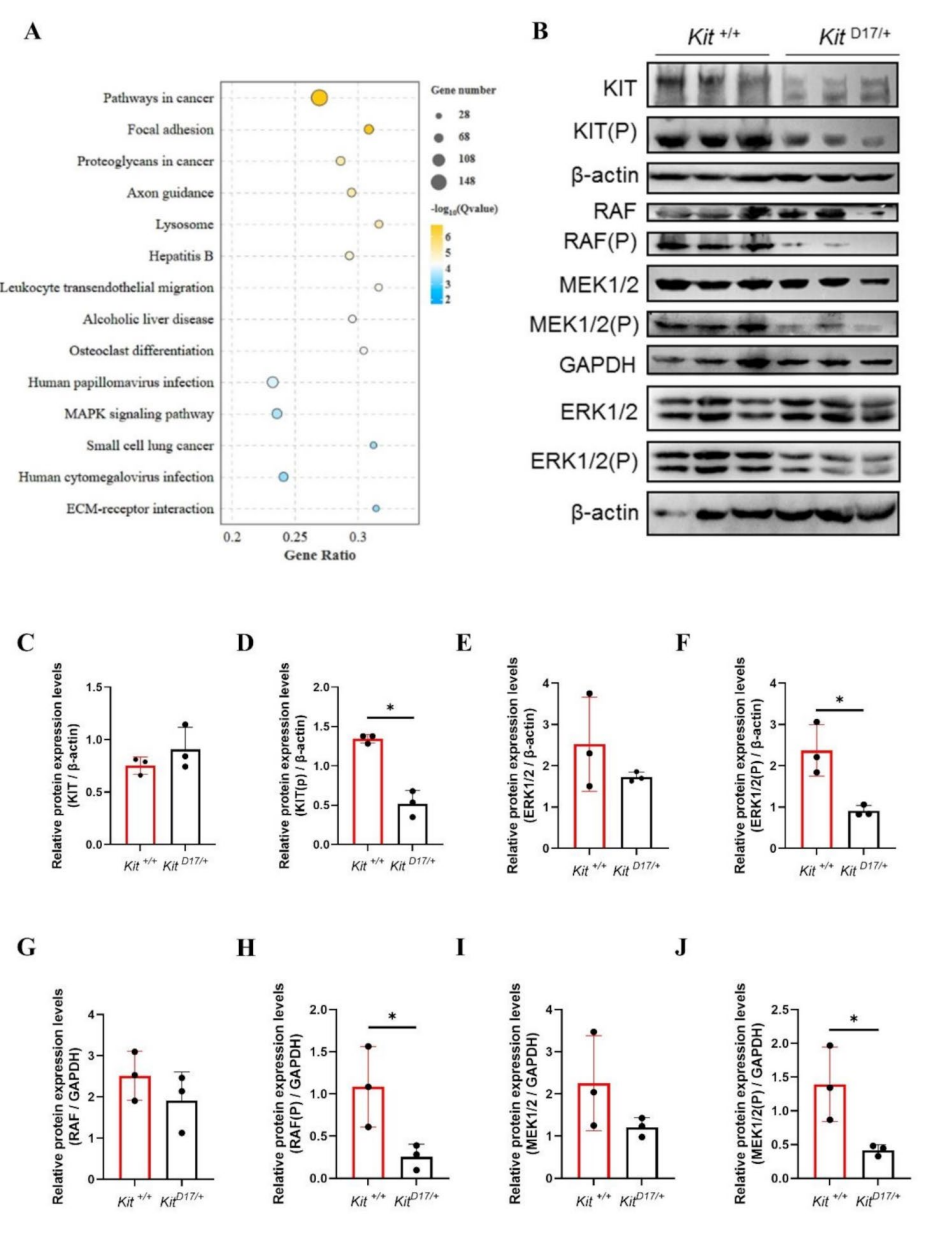
KEGG enrichment analysis and western blotting analysis. **(A)** Common signaling pathways in the top 30 enriched KEGG pathways of DEGs between testicular tissues of 5-week-old and 7-week-old *Kit*^*+/+*^ and *Kit*^*D17/+*^ mice. **(B)** Protein expression assay for KIT, KIT (P), RAF, RAF (P), MEK1/2, MEK1/2 (P), ERK1/2, and ERK1/2 (P) in *Kit* ^D17/+^ and *Kit* ^+/+^ testis tissue. β-actin and GAPDH served as loading controls, *n* = 3. **(C-J)** Quantification of protein expression for KIT, KIT (P), ERK1/2, ERK1/2 (P), RAF, RAF (P), MEK1/2, and MEK12 (P) based on the intensity of the blotting bands. The data are shown as the mean (± SEM) of three independent experiments. Student’s t test; **P < 0.01, *P < 0.05

KEGG enrichment analysis revealed that the MAPK signaling pathway, which is critical for cell differentiation and apoptosis, was significantly enriched in the 5 W and 7 W groups (Fig. 6A). Therefore, we speculated that the difference in spermatogonia differentiation between *Kit* ^*D17/+*^ and *Kit* ^+/+^ mice may be related to the MAPK signaling pathway. To verify whether deletion of exon 17 affects spermatogenesis via the MAPK cascade, western blotting was performed to determine the expression of downstream factors of the MAPK signaling pathway. The deletion of exon 17 led to a truncated intracellular kinase domain in the KIT protein, which we speculated may affect the phosphorylation level of KIT; therefore, we first examined the self-phosphorylation function of KIT. The results showed that there was no significant change in the expression of total KIT protein in testicular tissues between *Kit* ^*D17/+*^ and *Kit* ^*+/+*^ mice (Fig. 6B and C), whereas the expression of phosphorylated KIT decreased significantly in *Kit* ^*D17/+*^ mice (Fig. 6B and D). As impaired KIT phosphorylation may affect the activation of downstream signaling pathways, we proceeded to examine the expression levels of phosphorylated serine/threonine-protein kinase (RAF(P)), phosphorylated mitogen-activated protein kinase kinase 2 (MEK1/2(P)), and extracellular signal-regulated kinases 1 and 2 (ERK1/2(P)). We observed no significant changes in the expression of total RAF, MEK1/2 or ERK1/2 (Fig. 6B, E, G and I) but did observe notable reductions in RAF (P), MEK1/2 (P), and ERK1/2 (P) levels in *Kit* ^*D17/+*^ mice (Fig. 6B, F, H and J). In conclusion, our findings indicate that the deletion of exon 17 inhibits the phosphorylation of KIT, RAF, MEK1/2 and ERK1/2, which in turn may disrupt the differentiation of spermatogonia through the MAPK/ERK signaling cascade.

## Discussion

The dominant white phenotype in domestic pigs is caused primarily by complex structural mutations in the *KIT* gene, including a splice mutation at the first nucleotide of intron 17, which leads to the skipping of exon 17 [4–6]. Previous studies have indicated that, compared with Duroc pigs, dominant white pigs (Yorkshire pigs or Landrace pigs) exhibit smaller testes, reduced sperm motility and lower sperm density [17–19]. Through analysis of a mouse model with heterozygous deletion of exon 17 of *Kit*, we found that the splice mutation caused a range of reproductive phenotypic traits, including a smaller testis and epididymis and poor sperm quality and reproductive ability (Figs. 1 and 2), which are similar to the phenotype observed in dominant white pig breeds. Therefore, we consider that the less developed testes and lower sperm quality of the dominant white pigs should be attributed mainly to the splice mutation in *KIT*.

The histological analysis of testicular tissue sections revealed increased occurrence of lumen vacuolization and atrophy of the seminiferous epithelium in *Kit* ^*D17/+*^ mice (Fig. 1F-I), which was closely related to a lower sperm count and motility and lower male fertility in *Kit* ^*D17/+*^ mice (Fig. 2B-N). Our findings provide further evidence to support the speculation that the reduced sperm quality of dominant white pigs should be attributed to the splice mutation in porcine *KIT*.

The *Kit* gene plays multiple roles in spermatogenesis, including preventing apoptosis in primordial germ cells (PGCs), promoting cell replication in both PGCs and spermatogonia (Spg), and initiating the entry of Spg into meiosis [29]. Kit is specifically expressed in differentiated spermatogonia and is recognized as a marker for the transition from undifferentiated to differentiating type A spermatogonia [30]. Although the functions of the *Kit* gene in spermatogenesis have been increasingly documented, the specific effects of the splice mutation in the *KIT* gene of dominant white pigs on spermatogenesis have not been verified. Our study demonstrates that the deletion of exon 17 of *Kit* severely impaired the differentiation of spermatogonia, leading to a reduced number of Sycp^+^, Ddx4^+^ and Dazl^+^ cells, whereas this deletion did not affect the number of PGP9.5^+^ cells (Fig. 3). Our results are consistent with the findings of previous studies showing that monoallelic insertional inactivation and point mutations of *Kit* did not affect spermatogonia stem cell (SSC) maintenance but inhibited the progression of meiosis, resulting in a decrease in the number of differentiated spermatogonia [31, 32]. Therefore, the 41 amino acids of a highly conserved region of the tyrosine kinase domain containing the Tyr 823 residue, encoded by exon 17 of *Kit*, should be considered critical for *Kit* tyrosine kinase activity and signaling functions in spermatogenesis. In addition, we found that the splice mutation of *Kit* also affected cell survival, as TUNEL analysis revealed increased apoptosis of testicular cells in *Kit* ^*D17/+*^ mice (Fig. 4A), which may have contributed to the decrease in the number of germ cells and sperm in *Kit* ^*D17/+*^ mice.

The MAPK signaling pathway is activated by a variety of cell surface receptors and plays important roles in various cellular processes [33]. KIT-mediated cell signaling can activate multiple MAPKs, including extracellular-signal-regulated kinase (ERK)1/2, p38, c-Jun N-terminal kinase (JNK), and ERK5 [7, 34, 35]. Through transcriptomic analysis and western blot analysis, we found that the splice mutation of *Kit* impaired MAPK signaling functions, as evidenced by attenuated ERK1/2 signaling (Fig. 6B-I), which ultimately led to the downregulation of many genes involved in meiosis and spermatogenesis. These findings could provide new insights into the regulatory role of the splice mutation of *KIT* of spermatogenesis in dominant pigs.

Reproductive traits, including litter size and semen quality, are complex traits controlled by multiple gene regulatory pathways [36]. Although there is no direct example of improved semen quality through gene editing technology, one recent study has shown that gene editing has the potential to increase sperm quality in animals [37]. In one study, MSTN-edited cattle were created via CRISPR/Cas9, and the sperm motility parameters, including WOB (wobble) and BCF (beat cross-frequency) of the gene-edited cattle were significantly greater than those of their wild-type counterparts [37]. Therefore, we propose that correction of the splice mutation of *KIT* in dominant white pigs through gene editing technology, such as base editing, would restore the impaired *KIT* signaling functions involved in spermatogenesis and improve the semen quality of Yorkshire or Landrace pigs.

## Conclusions

In conclusion, our study demonstrated that the splice mutation of *Kit*, which causes the skipping of exon 17, severely impaired spermatogenesis and promoted the apoptosis of testicular cells through MAPK-ERK signaling, leading to lower semen quality and male fertility. These findings could provide a theoretical basis for improving the semen quality of dominant white pigs through correction of the splice mutation of KIT using gene editing technology.

## Electronic supplementary material

Below is the link to the electronic supplementary material.

Supplementary Material 1

Supplementary Material 2

## Data Availability

The datasets used and/or analyzed during the current study are available from the corresponding author upon reasonable request.

## Abbreviations

PGC: Primordial germ cells
Spg: Spermatogonia
SSCs: Spermatogonial stem cell
CASA: Computer Assisted Sperm Analysis
VAP: Average path velocity
VCL: Curvilinear velocity
DAP: Average path distance
DCL: Curvilinear distance
DSL: Straight line distance
VSL: Straight line velocity
ALH: Head displacement
BCF: Beat cross frequency
ERK: Extracellular-signal-regulated kinase
MAPK: The mitogen-activated protein kinase
JAK/STAT: The Janus kinase/signal transducers and activators of transcription
PI3-K: The phosphatidylinositol 3-kinase
