# Supplementary material for: Heterozygous deletion of exon 17 of the Kit gene impairs mouse spermatogenesis by attenuating MAPK-ERK signaling
Source: Biol Res. 2025 May 13;58:28. doi: 10.1186/s40659-025-00609-2 (PMC12070560; doi:10.1186/s40659-025-00609-2)
Supplement: Supplementary file 1 — Supplementary Material 1 [file 40659_2025_609_MOESM1_ESM.doc]

**Supplementary materials**

**
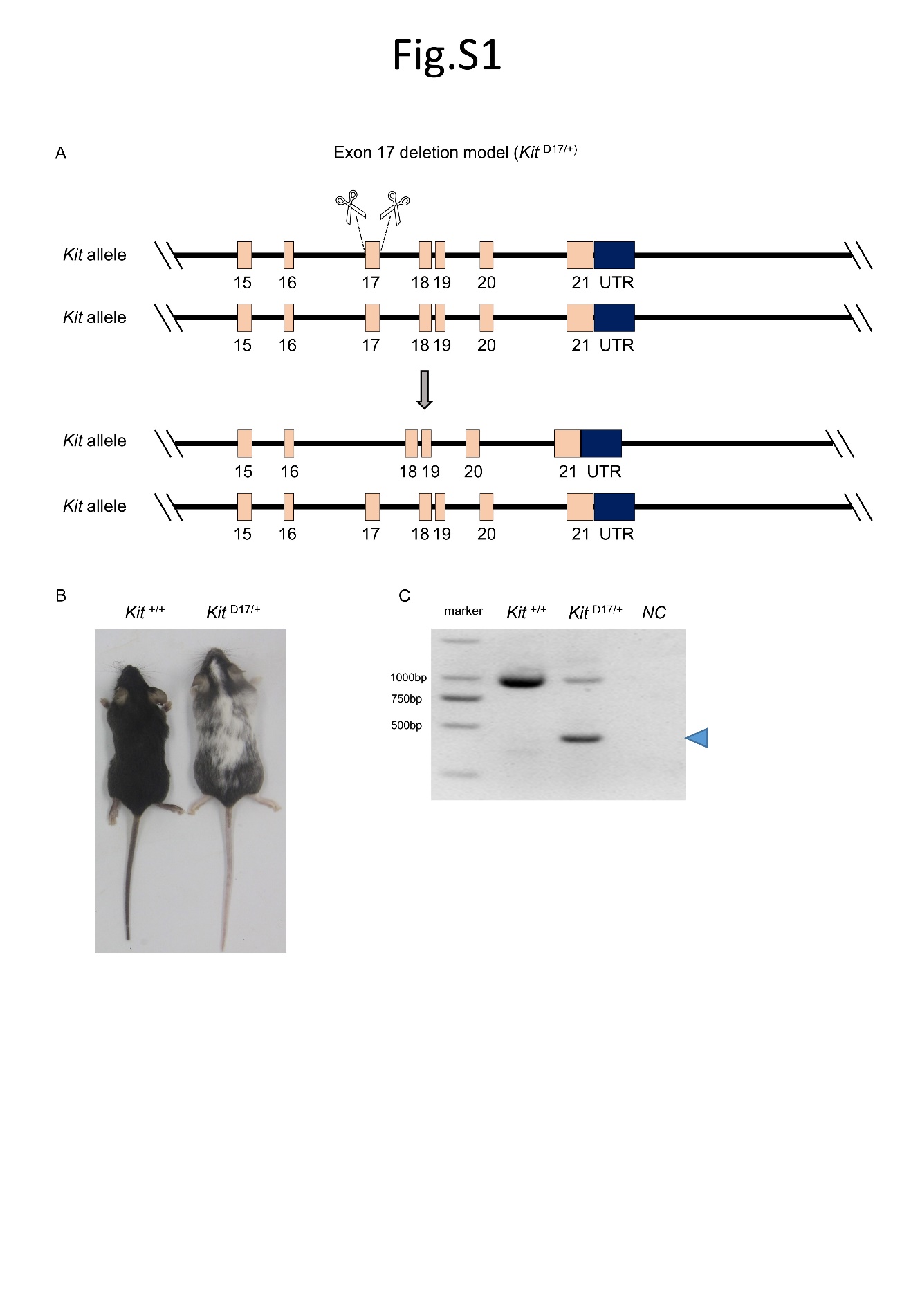
**

**Fig. S1.** Generation of *Kit* ^D17/+^mouse model. (A) Schematic diagram of creation of *Kit* ^D17/+^ mouse model by using CRISPR-Cas9. (B) The coat colour phenotype of *Kit* ^D17/+^ mouse. (C) PCR analysis of the deletion of exon 17 of *Kit* in *Kit* ^D17/+^ mouse.

**
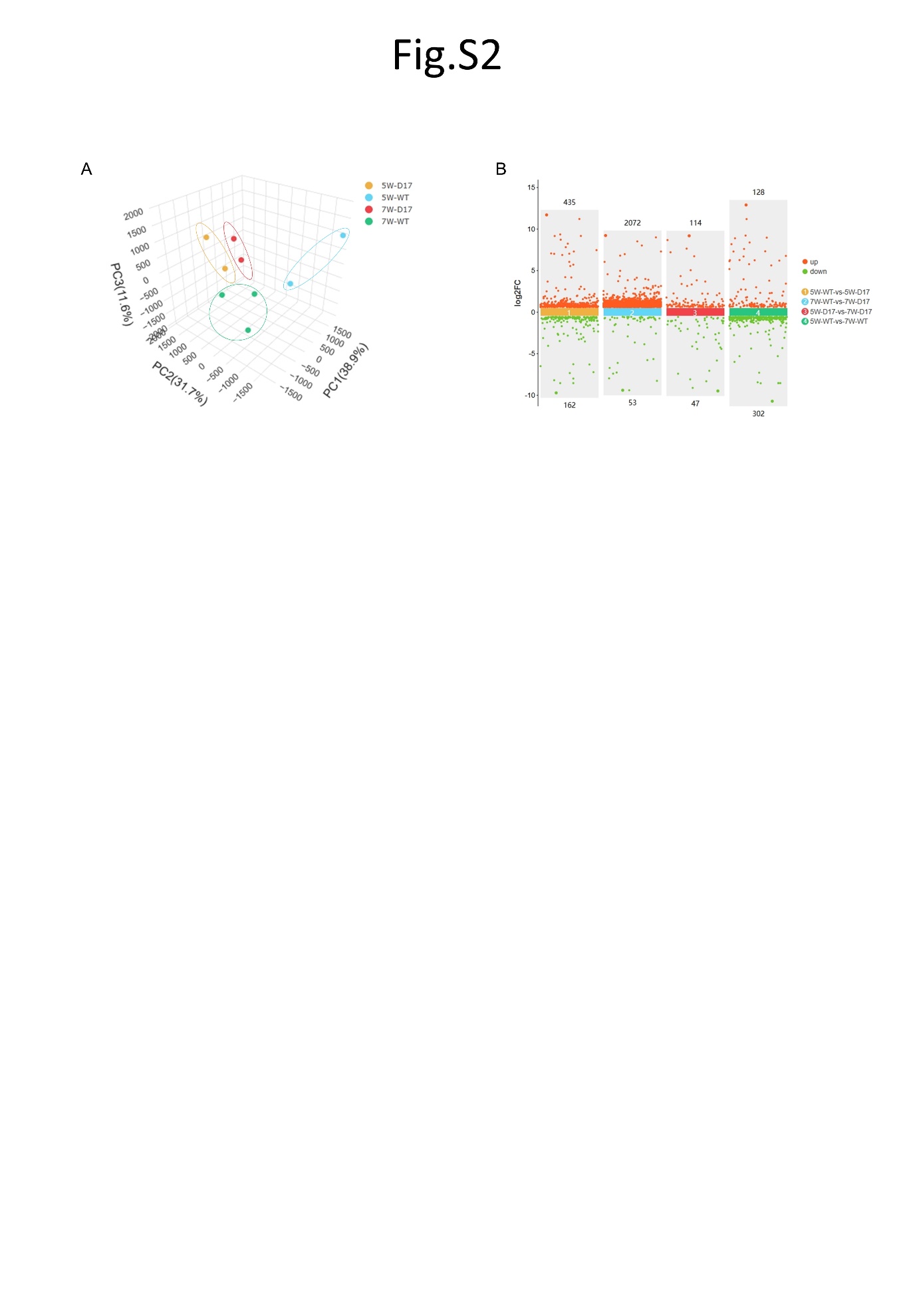
**

**Fig. S2.** Sample relationships and DEG analyses (A) Three-dimensional principal component analysis (PCA). (B) Number of up- and down-regulated genes in testicular tissues of mice at 5 and 7 weeks.

Table S1. Information of antibodies used in this study.

| Antibody | Application | Brands | Product code |
| --- | --- | --- | --- |
| Pgp9.5 | IF | Abcam | ab8189 |
| Dazl | IF | Abcam | ab34139 |
| Ddx4 | IF | ABclonal | A15624 |
| Kit | WB | Abcam | ab256345 |
| Kit（p） | WB | Abcam | ab62154 |
| Sox9 | IF | Merck | AB5535 |
| Sycp3 | IF | Abcam | ab97672 |
| Ki67 | IHC | Abcam | Ab15580 |
| β-actin | WB | ABclonal | AC026 |
| Erk1/2 | WB | CST | 4695T |
| Erk1/2(p) | WB | CST | 9101S |
| Goat Anti-Mouse IgG HRP | WB | Abmart | M21001F |
| Goat Anti-Rabbit IgG-HRP | WB | Abmart | M21002F |
| Alexa Fluor® 488 | IF | CST | 4412S |
| Alexa Fluor® 594 | IF | Abcam | ab150080 |
| Alexa Fluor® 555 | IF | CST | 4409S |

Antibodies were diluted in TBST containing 5% BSA. The dilution rate of each antibody was adjusted according to the manufacturer’s instructions and the requirements for optimization of the experimental procedures.

Table S2. Primers used for RT-qPCR analysis.

| Primer name | Sequence（5’-3’） |
| --- | --- |
| *SYCP3* F  *SYCP3* R | GAAATCTGGGAAGCCACCTT  GCTCCAAATTTTTCCAGCAT |
| *DMC1* F  *DMC1* R | GGCATTTTCAAACTATTGATCG  GATCAGCCTGGAAGGTCATA |
| *MSH4* F  *MSH4* R | TCCGCGCCAGGTTCATCATTTG  TTTATGCCCTGATCTCTGGGGA |
| *CCNB1IP1* F  *CCNB1IP1* R | TGTTGCAGGGTTCTTTTGGAGAT  TCGCTGGTGAACGACTGAACTC |
| *HORMAD1* F  *HORMAD1* R | GCTTCCCTGAGTGCATTGGTATT  GCATCCACTTCACTAGCTGTGAA |
| *MEIOB* F  *MEIOB* R | GGGCTGCACGATAAATGAGTTTC  TCCTGCTTGCCTCAGTAGGATC |
