# Supplementary material for: Heterozygous deletion of exon 17 of the Kit gene impairs mouse spermatogenesis by attenuating MAPK-ERK signaling
Source: Biol Res. 2025 May 13;58:28. doi: 10.1186/s40659-025-00609-2 (PMC12070560; doi:10.1186/s40659-025-00609-2)
Supplement: Supplementary file 2 — Supplementary Material 2 [file 40659_2025_609_MOESM2_ESM.pdf]

Editing Certificate

This document certifies that the manuscript

Heterozygous deletion of exon 17 of the Kit gene impairs mouse spermatogenesis by attenuating MAPK-ERK signaling

prepared by the authors

Siyuan Lin, Min Yang, Weipeng Zhu, Changqi Yang, Yaosheng Chen, Peiqing Cong, Xiaohong Liu, Zuyong He

was edited for proper English language, grammar, punctuation, spelling, and overall style by one or more of the highly qualified English speaking editors at AJE.

This certificate was issued on **March 5, 2025** and may be verified on the AJE website using the verification code **73FE-9066-E233-EE18-95D8**.

Neither the research content nor the authors' intentions were altered in any way during the editing process. Documents receiving this certification should be English-ready for publication; however, the author has the ability to accept or reject our suggestions and changes. To verify the final AJE edited version, please visit our verification page at aje.com/certificate. If you have any questions or concerns about this edited document, please contact AJE at support@aje.com.

AJE provides a range of editing, translation, and manuscript services for researchers and publishers around the world.
For more information about our company, services, and partner discounts, please visit aje.com.
